# Treatment for Retinopathy of Prematurity in an Infant with Adenoviral Conjunctivitis

**DOI:** 10.1155/2015/192717

**Published:** 2015-03-19

**Authors:** Murat Gunay, Gokhan Celik, Rahim Con

**Affiliations:** ^1^Zeynep Kamil Maternity and Children's Diseases Training and Research Hospital, Department of Ophthalmology, 34668 Istanbul, Turkey; ^2^Sanliurfa Obstetrics and Gynecology Hospital, Department of Ophthalmology, 63050 Sanliurfa, Turkey

## Abstract

Retinopathy of prematurity (ROP) has been a major problematic disorder during childhood. Laser photocoagulation (LPC) has been proven to be effective in most of the ROP cases. Adenoviral conjunctivitis (AVC) is responsible for epidemics among adult and pediatric population. It has also been reported to be a cause of outbreaks in neonatal intensive care units (NICU) several times. We herein demonstrate a case with AVC who underwent LPC for ROP. And we discuss the treatment methodology in such cases.

## 1. Introduction

Retinopathy of prematurity (ROP) has been a leading cause of childhood blindness in developed and developing countries worldwide [1]. Cryotherapy, laser photocoagulation (LPC), and, later on, intravitreal bevacizumab injection (IVB) were administered for the treatment of the disease. Although LPC was the mainstay treatment option for most of ROP cases, IVB therapy has provided us with an alternative modality of treatment [2–4].

Adenoviral conjunctivitis (AVC), an acute ocular infection, includes findings such as photophobia, conjunctival injection, and excessive lacrimation [5]. The common form of AVC is epidemic keratoconjunctivitis which is responsible for several outbreaks among adult and pediatric population [6–8]. Various studies reported outbreaks due to adenovirus infections at neonatal intensive care unit (NICU). Also the association between AVC and ROP examinations has been previously published [9–11].

Our aim in this study was to introduce our clinical approach in LPC for ROP in a premature neonate infected with AVC.

## 2. Case Report

**Figure 1:**
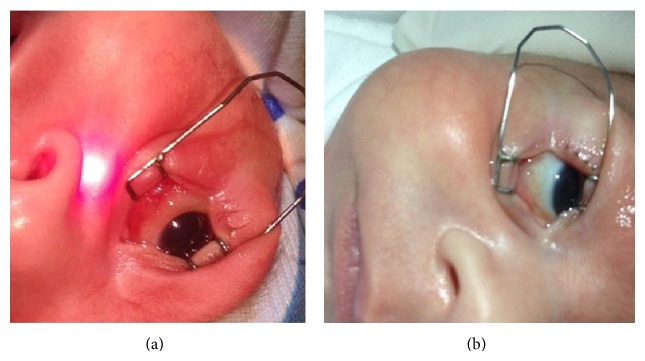
Edema of the inferior fornix conjunctiva and chemosis are easily seen before the first laser session in the right eye of the infant (a). Conjunctivitis is mostly resolved after medical therapy before the second laser session in the same eye (b).

An infant with gestational age (GA) of 32 weeks and birth weight (BW) of 1440 g was firstly examined on postnatal 36 weeks for routine ROP screening in NICU of Zeynep Kamil Maternity and Children's Diseases Training and Research Hospital. Stage 1 zone II ROP without plus disease was noted in both eyes on fundus examination according to the International Classification of Retinopathy of Prematurity (ICROP) at first visit [12]. The child showed no other abnormalities in anterior and/or posterior segment. Five days later, the child presented with periorbital edema, redness, and tearing for which an ophthalmology consult was sought. There were 6 other infants at the same unit who presented with the same signs at the same time. The diagnosis of presumed AVC was made and the same medication was ordered for all affected neonates including topical antibiotic drops, artificial tear drops, and conjunctival irrigation with diluted povidone iodine. Because of not performing any laboratory investigations for the detection of adenovirus antigen, the diagnosis of presumed AVC was made empirically in all infants who had the same findings. All children who suffered from AVC were isolated in another room in NICU. Weekly examinations were performed for AVC during the follow-up period. Our case developed bilateral stage 3 zone II ROP with plus disease at postnatal 39 weeks. The child was still showing conjunctival chemosis, mild eyelid edema, pseudomembrane formation at tarsal conjunctiva, and mild corneal edema in both eyes; see Figure 1.

Laser treatment was considered in order to prevent the progression of ROP. A detailed informed consent was taken from the parents. 810 nm diode laser device (Iridex; Oculight SL, Mountainview, CA, U.S.A) was used for laser ablation. The laser was delivered to the avascular retina anterior to the ridge and posterior to the ridge which involved higher amount of fibrovascular component. Numbers of laser spots applied to right and left eyes were 620 and 605, at first laser session, respectively. LPC session ended due to increased conjunctival chemosis, conjunctival hemorrhage, and clouding of the ocular media. Topical drops were continued. And five days after the first laser session, the child was subjected to a second laser session to complete the ablation of the rest of avascular retinal zones. Totally 356 and 325 laser spots were delivered at second laser session, respectively.

The infant showed total recovery from AVC one week after the second laser session (Figure 1). ROP began to resolve and regressed after 3 weeks to a favorable outcome.

## 3. Discussion

There are several types of adenovirus infection in neonates including the most frequent ones as 4, 8, 11, 19, and 37. Outbreaks due to AVC in NICU centers remain an important comorbid factor particularly in infants with treatment requiring ROP [13]. As far as we know there was no information existing about the treatment approach in such cases in previous literature. Therefore, our aim in this report was to introduce a ROP case infected with AVC and discuss our treatment modality.

It has been previously stated that outbreaks of AVC in NICU were commonly manifested after ophthalmological examination procedures due to contaminated instruments [9, 11]. Low BW and patient care factors were also shown to be other causes of conjunctivitis among preterm infants [14]. Totally 7 newborns had infection with AVC in NICU during the study period and the current case was affected by the spread of this infection. Disposable types of equipment (eyelid speculum and depressor) are routinely used in our clinic for ROP examinations. However, several other factors such as inadequate hand hygiene, not excluding the infected staff members in NICU, and late isolation procedures of the infected infants in the same unit might also contribute to the spread of AVC [10].

Treatment requiring ROP can lead to blindness without timely intervention. Laser photocoagulation has been shown to be useful in ROP management. It prevents the progression of the disease and results in favorable outcomes [15]. The ETROP study indicated LPC for infants who had a high risk of progression for the disease [3]. Our newborn had stage 3 zone II high-risk prethreshold ROP at the time of the intervention as well as AVC related ocular findings. Laser application of the vascular retina posterior to the proliferative ridge tissue has been reported to lead an easier regression of ROP with favorable outcomes [15–17]. We performed a similar pattern of LPC in our case. However, we could not proceed to treatment due to increased chemosis of conjunctiva, hemorrhage from conjunctival membranes, and corneal haze in both eyes. We halted the procedure and rearranged a second laser session in order to complete laser ablation of the remaining avascular zones. Although divided laser sessions were mostly reported in aggressive posterior ROP (APROP) cases due to inadequate regression of the disease [18], gradual worsening of ocular surface findings in the present case compelled us to perform LPC in two laser sittings. Actually, this seemed to be an effective method in our case.

Several topical treatment methods have been applied for AVC, most of which are prophylactic including preservative-free antibiotic and artificial tears. Also some authors found povidone iodine 2.5% as an effective treatment in AVC [19]. We used the same treatment methodology during the course of AVC. And it resolves without any sequelae on ocular surface.

In conclusion, viral conjunctivitis related ocular surface findings may prevent laser ablation in a classical ROP case. Divided sessions of laser treatment may be an option in such cases. Also a more experienced surgeon in ROP treatment could handle the situation much more easily as well. Furthermore, immediate and careful treatment must be carried out for AVC conjunctivitis in an infant who has possibility for progression to treatment required ROP. Neonates with ROP and those who are infected with AVC in NICU should be subjected to immediate topical medication in order to perform an adequate LPC procedure.
